# Impact of CYP2D6*2, CYP2D6*35, rs5758550, and related haplotypes on risperidone clearance in vivo

**DOI:** 10.1007/s00228-024-03721-6

**Published:** 2024-07-04

**Authors:** Elisabet Størset, Line Skute Bråten, Magnus Ingelman-Sundberg, Inger Johansson, Espen Molden, Marianne Kristiansen Kringen

**Affiliations:** 1 Center for Psychopharmacology, Diakonhjemmet Hospital, Oslo, Norway; 2 Section of Pharmacogenetics, Department of Physiology and Pharmacology, Karolinska Institutet, Stockholm, Sweden; 3 Department of Pharmacy, University of Oslo, Oslo, Norway; 4 Department of Life Science and Health, Oslo Metropolitan University, Oslo, Norway

**Keywords:** Pharmacogenomics, CYP haplotypes, Pharmacokinetic modelling, Precision dosing

## Abstract

**Purpose:**

The *CYP2D6* gene exhibits significant polymorphism, contributing to variability in responses to drugs metabolized by CYP2D6. While CYP2D6*2 and CYP2D6*35 are presently designated as alleles encoding normal metabolism, this classification is based on moderate level evidence. Additionally, the role of the formerly called “enhancer” single nucleotide polymorphism (SNP) rs5758550 is unclear. In this study, the impacts of CYP2D6*2, CYP2D6*35 and rs5758550 on CYP2D6 activity were investigated using risperidone clearance as CYP2D6 activity marker.

**Methods:**

A joint parent-metabolite population pharmacokinetic model was used to describe 1,565 serum concentration measurements of risperidone and 9-hydroxyrisperidone in 512 subjects. Risperidone population clearance was modeled as the sum of a CYP2D6-independent clearance term and the partial clearances contributed from each individually expressed *CYP2D6* allele or haplotype. In addition to the well-characterized *CYP2D6* alleles (*3-*6, *9, *10 and *41), *2, *35 and two haplotypes assigned as CYP2D6*2-rs5758550G and CYP2D6*2-rs5758550A were evaluated.

**Results:**

Each evaluated *CYP2D6* allele was associated with significantly lower risperidone clearance than the reference normal function allele CYP2D6*1 (p < 0.001). Further, rs5758550 differentiated the effect of CYP2D6*2 (p = 0.005). The haplotype-specific clearances for CYP2D6*2-rs5758550A, CYP2D6*2-rs5758550G and CYP2D6*35 were estimated to 30%, 66% and 57%, respectively, relative to the clearance for CYP2D6*1. Notably, rs5758550 is in high linkage disequilibrium (R^2^ > 0.85) with at least 24 other SNPs and cannot be assigned as a functional SNP.

**Conclusion:**

CYP2D6*2 and CYP2D6*35 encode reduced risperidone clearance, and the extent of reduction for CYP2D6*2 is differentiated by rs5758550*.* Genotyping of these haplotypes might improve the precision of genotype-guided prediction of CYP2D6-mediated clearance.

**Supplementary Information:**

The online version contains supplementary material available at 10.1007/s00228-024-03721-6.

## Introduction

Cytochrome P450 2D6 (CYP2D6) is involved in the biotransformation of about 25% of clinically used drugs [1]. CYP2D6 activity varies considerably between subjects, leading to substantial differences in systemic exposure and clinical response to standard doses of drugs metabolized by CYP2D6 [2]. Genetic polymorphism is one of the main sources of interindividual differences in CYP2D6 activity. Many of the genetic variant alleles have been found to encode increased, reduced, or deficient CYP2D6 activity and are included in genotyping panels to predict individual dose requirements [3]. However, the functional impact of many of these variants or haplotypes on CYP2D6 activity remains unclear. A better understanding of both known and novel variants or haplotypes affecting CYP2D6 activity would improve genotype-guided dosing and potentially clinical outcomes in patients treated with CYP2D6-metabolized drugs.

CYP2D6*2 and CYP2D6*35 are currently classified as normal functional alleles with similar effects on enzyme activity as CYP2D6*1 [3, 4]. However, there are several studies on various CYP2D6 substrates (including tamoxifen, dextromethorphan, carvedilol, and brexpiprazole) suggesting that CYP2D6*2 is associated with reduced CYP2D6 activity [5–9], which calls into question the currently proposed functional assignment of CYP2D6*2. However, no such effect was observed with other CYP2D6 substrates such as vortioxetine and tedatioxetine [10, 11], which may indicate that CYP2D6*2 has a substrate-dependent effect on enzyme activity as also indicated from previous in vitro studies [12]. The fact that CYP2D6*2 is a Tier 1 allele recommended for routine clinical testing [13] emphasizes the need for further studies to ensure correct functional annotation.

Previously, much attention has been taken to the role of the so-called “enhancer” single nucleotide polymorphism (SNP) rs5758550 G>A ~ 115 kb downstream of the *CYP2D6* gene in regulating *CYP2D6* gene expression [14–17], particularly for CYP2D6*2 alleles. The* G* variant of this SNP has been hypothesized to increase *CYP2D6* transcription and contribute to interindividual variability in CYP2D6 activity, but other research groups have not succeeded in reproducing the “enhancer” effect [18, 19]. In a recent review of in vivo studies on the rs5758550 variant, it was concluded that there is currently insufficient evidence to support the use of rs5758550 in the clinic due to the methodological caveats of the studies and limited understanding of underlying mechanism [17].

Regarding CYP2D6*35, the assigned classification of normal function is based on moderate level evidence from a limited number of studies [4]. It was originally hypothesized that this variant encodes for increased enzyme activity [20], but these results could not be reproduced in vitro and in vivo. More recent results have been inconsistent as to whether CYP2D6*35 encodes for normal [6, 21] or even reduced enzyme activity [8, 12, 22].

To obtain a measure of CYP2D6-mediated clearance in the present study, we used the population pharmacokinetic modelling approach on a large sample of parent-metabolite serum concentration data for risperidone, which is metabolized primarily by CYP2D6 with a rate known to be dependent on *CYP2D6* genotype [23, 24]. The aim of the study was to quantify the influence of several genetic variants or haplotypes on CYP2D6-mediated clearance, with a specific focus on CYP2D6*2, CYP2D6*35, and rs5758550.

## Methods

### Subjects and data

The included subjects were above the age of 18 years with known *CYP2D6* genotype and a history of therapeutic drug monitoring (TDM) of risperidone and 9-hydroxyrisperidone serum concentrations following oral administration of risperidone. Data were collected from routine clinical analyses of patient samples performed at the Center for Psychopharmacology, Diakonhjemmet Hospital (Oslo, Norway) between 2005 and 2022.

### Dosing regimen and pharmacokinetic sampling

The TDM requisition forms filled out by the treating physician specified information on dosing history and time between last dose and blood sampling. Samples were considered only if this information was clearly provided. The requisition form suggests blood sampling 12 to 24 h after the last dose as recommended for antipsychotics [25]. To increase the sample size, samples taken between 9 and 30 h after the last dose were eligible for inclusion. Samples were included regardless of dose and dosing frequency (i.e., once- or twice-daily risperidone) as the individual dosing history was specified in the dataset for pharmacokinetic analysis. The TDM samples were assumed to be taken at steady state unless otherwise recorded on the requisition forms.

Samples were excluded if the requisition form indicated concomitant use of the CYP2D6-inhibiting drugs paroxetine, fluoxetine, or bupropion or the CYP3A-inducing drugs phenobarbital, phenytoin, or carbamazepine, or if long-acting formulations of risperidone or 9-hydroxyrisperidone (paliperidone) had been injected during the last 9 months.

### Serum concentration analysis

Serum concentration analysis of risperidone and 9-hydroxyrisperidone was carried out as part of the routine therapeutic drug monitoring service at the Center for Psychopharmacology. The method was based on ultrahigh-performance liquid chromatography high-resolution mass spectrometry (UHPLC-HRMS). Briefly, serum samples were purified by protein precipitation mixing 200 μL of serum aliquot with 400 μL of acetonitrile-methanol (90/10 vol/vol), which included the isotope-labelled internal standards. Following centrifugation, the supernatants were diluted 1:1 with ultrapure water, and 4 μL of purified sample was then injected into a Vanquish Binary UHPLC system coupled to a Q Exactive Orbitrap HRAM MS with electrospray ionization operated in positive ionization mode (Thermo Scientific, Waltham, MS, USA). Chromatographic separation was performed on a XBridge BEH C18 column (2.5 μm, 2.1 × 75 mm; Waters). The mobile phase gradient comprised a mixture of acetonitrile and ammonium acetate buffer (pH 4.8).

All the calibration curves were linear (*R*^2^ > 0.99) in validated ranges: risperidone, 1–200 nmol/L; 9OH-risperidone, 2.5–300 nmol/L. Imprecision and inaccuracy parameters of the assays were lower than 15%. The lower limit of detection was 0.4 nM for risperidone and 1 nM for 9-hydroxyrisperidone, and concentrations above this limit were reported and available for pharmacokinetic modelling analysis. During population modelling, values below the detection limit were handled by imputing the half of the lower limit of detection due to a proportion of < 10% [26, 27].

### Pharmacogenetic analyses

Genotyping of *CYP2D6* had previously been performed by TaqMan-based real-time PCR assays implemented for routine pharmacogenetic analysis at the Center for Psychopharmacology. The routine panel for *CYP2D6* genotyping included the null alleles CYP2D6*3 (rs35742686), CYP2D6*4 (rs3892097), CYP2D6*5 (whole gene deletion), and CYP2D6*6 (rs5030655) and the reduced function alleles CYP2D6*9 (rs5030656), CYP2D6*10 (rs1065852), and CYP2D6*41 (rs28371725), as well as copy number analysis to identify multiplication of the *CYP2D6* gene giving rise to ultrarapid metabolism.

In addition to *CYP2D6* variant alleles, other gene variants or haplotypes may also affect risperidone clearance, including a variant in the gene encoding the nuclear factor I B (NFIB), which has been shown to regulate the expression of the *CYP2D6* gene [28, 29], and CYP3A4*22, which may alter CYP3A4-mediated risperidone clearance [30].

To get a better coverage of variant alleles that may affect risperidone clearance, the DNA samples were reanalyzed with predesigned TaqMan-based real-time PCR assays (Thermo Fisher Scientific, Waltham, MA, USA) to detect CYP2D6*2 (rs16947; C__27102425_10), CYP2D6*35 (rs769258; C__7102444_F0), rs5758550 (C_29692254_10), *NFIB* (rs28379954; C_59359617_10), and CYP3A4*22 (rs35599367; C__59013445_10). Subjects carrying CYP2D6*2 and rs5758550 *G* and *A* were defined as carriers of haplotypes CYP2D6*2-rs5758550G and CYP2D6*2-rs5758550A, respectively.

### Exploratory data analysis

Initially, the metabolic ratios (MR) between the measured 9-hydroxyrisperidone and risperidone concentrations were calculated at each observation and considered a raw data-based approximation of enzyme activity. The relationship between the various CYP2D6 diplotypes and the median MR for each subject was visually explored using box plots and by pairwise comparisons using the Mann–Whitney *U* test. Any apparent relationship between CYP2D6 allele or haplotype and risperidone clearance was then quantified using population pharmacokinetic modelling of all data observations.

### Population pharmacokinetic modelling

The time courses of risperidone and 9-hydroxyrisperidone serum concentrations were analyzed using population pharmacokinetic modelling (i.e., non-linear mixed effects modelling). The model was developed to simultaneously describe the pharmacokinetics of the parent and the metabolite and to quantify parameter variability and covariate effects.

#### Structural and stochastic model

The structural model consisted of one compartment for risperidone (parent) and one compartment for 9-hydroxyrisperidone (metabolite), both with linear elimination. Due to the sparsely sampled data, additional compartments were not considered. The absorption of risperidone into the first compartment was described using a first-order absorption rate constant fixed to a previously reported value of 2.01 h^−1^ [31]. As only data after oral administration were available, the oral bioavailability (F) of risperidone and the fraction of risperidone converted into 9-hydroxyrisperidone (*f*_met_) were not identifiable, and the reported disposition parameters of the parent and metabolite are therefore reported as apparent values (e.g. CL/F, CL_met_/[F × *f*_met_]) [32].

Between-subject variability (BSV) was estimated for the clearance parameter of both the parent and metabolite using exponential models:

$${\text{CL}}_{i}=\text{TVCL} \times {e}^{\eta_{iCL}}$$

where CL_*i*_ is the risperidone or 9-hydroxyrisperidone clearance for the *i*th individual, and η_*i*CL_ denotes the difference between individual and population typical value (TVCL), which was assumed normally distributed with mean zero and variance ω_CL_^2^. The correlation between the individual clearances of risperidone and 9-hydroxyrisperidone was also estimated, while no BSV was estimated for the volume of distribution parameters due to the sparse sampling design. BSVs are reported as coefficients of variations, calculated as the square root of e^(ω2−1)^. The residual variability in concentration measurements (one submodel for each analyte) was initially modeled using combined additive and proportional error structures:

$${\text{Obs}}_{ij}={\text{Pred}}_{ij} \left(1+ {\varepsilon 1}_{ij}\right)+ {\varepsilon 2}_{ij}$$

where Obs_*ij*_ is the *j*th observed concentration in the *i*th individual, Pred_*ij*_ is the corresponding model prediction, and ε1 and ε2 are random error terms with means of zero and variances of σ1^2^ and σ2^2^, respectively. The residual error was considered simplified into proportional or additive submodels based on parameter estimate values and parameter significance levels.

#### Covariate model

To evaluate the effect of the various *CYP2D6* genotypes on risperidone clearance (CL), the clearance of risperidone was modeled as the sum of a CYP2D6-independent clearance term (base clearance: CL_base_) and the estimated clearances attributable to each of the *CYP2D6* alleles determined for the subject:

$${\text{CL}}_{\text{ risperidone}}= {\text{CL}}_{\text{base}}+ {\text{CL}}_{\text{CYP}2\text{D}6\text{ allele }1}+ {\text{CL}}_{\text{CYP}2\text{D}6\text{ allele }2}$$

As an example, the total risperidone clearance for a subject with *CYP2D6* diplotype of *1/*35 was predicted by the sum of the estimated parameters CL_base_, CL_CYP2D6*1_, and CL_CYP2D6*35_. Allele-specific clearances for CYP2D6*2 (subsequently subdivided into new haplotypes based on rs5758550*A/G*), *9, *10, *35, and *41 relative to CYP2D6*1 were estimated, while the contribution to risperidone clearance from the deficient CYP2D6 alleles *3, *4, *5, and *6 was a priori fixed to zero. Further, *NFIB* genotype was tested as a covariate on total clearance or CYP2D6-mediated clearance, and gene variation in *CYP3A4* was tested as a covariate on the base clearance term (which is expected to be primarily mediated by CYP3A4). Heterozygous and homozygous carriers of CYP3A4*22 and the *NFIB-C* variant allele were merged due to minor proportions of homozygous carriers. In cases of unknown *CYP3A4* or *NFIB* genotype, the unknown genotype was tested either as a distinct covariate category or grouped together with the wildtype genotype.

In addition to gene variation, sex and age were evaluated as covariates on the clearance of risperidone. Sex and age were also evaluated on volumes of distributions and clearance of 9-hydroxyrisperidone, which has primarily renal elimination and is not a substrate for the enzymes under evaluation. Categorical covariates were evaluated by estimating the relative change in the pharmacokinetic parameter compared with the reference group. The impact of continuous covariates (i.e., patient age) was initially visually explored by estimating the relative change in the pharmacokinetic parameter of interest across age bins defined as 18–29 years, 30–39 years, 40–49 years, and so on. The relationship was then described in the final model using a mathematical function mimicking the observed relationship with age as a continuous covariate, such as linear, exponential, power, or piecewise linear functions.

The assessment of covariate relationships was undertaken using the forward inclusion and backward elimination procedure, which involves stepwise including the covariate with largest improvement in model fit for each round of covariate search until no more covariates significantly improve the model fit [33]. During the inclusion of covariates, *p* < 0.05 was used as the significance level, while during the backward elimination of covariates from the full covariate model, a more stringent significance level of 0.01 was required for the covariate to be retained in the final model.

#### Model evaluation

Model selection was primarily based on the differences in objective function value (ΔOFV), where ΔOFV of ≥ 3.84 when adding one parameter corresponds to a *p*-value of < 0.05 (χ^2^ distribution, 1 degree of freedom). Models were also evaluated by inspecting standard goodness-of-fit plots (observed vs. predicted concentrations and conditional weighted residuals vs. predicted concentrations and time after dose), prediction-corrected visual predictive checks (pcVPCs, generated from 1000 simulations) [34], biological plausibility of the parameter estimates and parameter uncertainty (95% confidence intervals derived from 5000 non-parametric bootstrap replicates) [35]. Finally, the observed metabolic ratios that were used for the initial exploratory analysis were overlaid with the model-predicted typical metabolic ratio in each *CYP2D6* diplotype group.

#### Software and estimation method

Population pharmacokinetic modelling was performed using the non-linear mixed effects modelling software NONMEM (v. 7.5.1, ICON Development Solutions, Hanover, MD, USA) with the first-order conditional estimation method with interaction (FOCE-I). Piraña [36] was used as graphical user interface. Data management, model evaluation, and graphical assessments were assisted by the R software, v. 4.2.1 [37], and the Perl-Speaks-NONMEM (PsN) toolkit [38].

### Haplotype analysis

Variants in high linkage disequilibrium (LD) with rs5758550 were assessed using LD-link [39]. LD-link is based on whole-genome sequence (WGS) data from 2504 individuals in the 1000 Genomes Project. The European cohort (EUR *n* = 503, i.e., 1,006 alleles) was used as a reference population for the haplotype assessment in this study. Variants with high LD (*R*^2^ values > 0.85) together with the SNPs identifying CYP2D6*2, CYP2D6*35, and CYP2D6*41 (rs16947, rs1135840, rs769258 (*35), rs28371725 (*41)) were used for haplotype analysis.

## Results

### Subjects and data

A total of 525 subjects were initially eligible for inclusion. Among these, 13 subjects were excluded due to inconclusive *CYP2D6* haplotypes (seven carriers of CYP2D6*1/*2-rs5758550G x N with unknown duplicated allele, and six carriers of CYP2D6*2/*5 and rs5758550 *A/G*). This left 512 subjects and 1026 alleles for analysis (Table 1 and Supplementary Table S1). The allele frequencies of CYP2D6*2-rs5758550A, CYP2D6*2-rs5758550G, and CYP2D6*35 were 3% (*n* = 27), 16% (*n* = 166), and 5% (*n* = 54), respectively. Only three subjects carried CYP2D6*1-rs5758550G. These alleles were therefore merged with CYP2D6*1-rs5758550A (*n* = 398). The included subjects contributed a total of 1565 serum measurement pairs of risperidone and 9-hydroxyrisperidone.

**Table 1:** Subject and sample characteristics

**Characteristic**	**Median (range) or number**
Sex	
Male, ***n***	275
Female, ***n***	237
Age, years; median (range)	37 (18–89)
Risperidone daily dose (mg); median (range)	3 (0.5–12)
Risperidone and 9-hydroxyrisperidone measurements	
In total, ***n***	1565
Per subject; median (range)	2 (1–41)
Risperidone dosing frequency prior to sampling, ***n***	
One dose per day	789
Two doses per day	758
Three doses per day	18
Time between dose and sampling, hours; median (range)	13 (9–30)
*CYP2D6* allele, ***n***	
*1	401^*^
*35	54
*2-rs5758550*A*	27
*2-rs5758550*G*	166^*^
*9	20
*10	23
*41	66
*3, 4, *5*, or *6* (deficient alleles)	269
*CYP3A4* allele, ***n***	
*1	939
*22	27
Not determined	58
*NFIB* allele, ***n***	
*T*	965
*C*	53
Not determined	6

*Two subjects contributed 3 alleles (i.e., *1/*1 xN, *n* = 1 and *2-rs5758550G/*2-rs5758550*G xN*, *n* = 1)

### Exploratory analysis

The initial exploratory analysis of the relationship between *CYP2D6* diplotypes and risperidone metabolic ratios (MR) indicated that each of the *CYP2D6* alleles under investigation was associated with reduced MR compared with the CYP2D6*1 reference allele (Supplementary Table S1 and Supplementary Fig. S1). Of particular interest, the observed MRs were generally lower in subjects carrying the CYP2D6*2-rs5758550G, CYP2D6*2-rs5758550A, or CYP2D6*35 haplotypes/alleles compared with CYP2D6*1. This encouraged further modelling of the data to quantify and statistically compare each allele’s impact on risperidone clearance.

### Population pharmacokinetic model

#### Structural and stochastic model

The pharmacokinetics of risperidone and its main metabolite 9-hydroxyrisperidone were adequately described using the one-compartment disposition models. Between-subject variabilities in their clearances were estimated to be 125% and 53%, respectively, prior to the inclusion of covariates. The residual error was described by a combined proportional and additive model for risperidone, while a proportional error model was sufficient for 9-hydroxyrisperidone (additive component estimated close to zero with no significant increase in OFV upon removal).

#### Covariate model

The inclusion of *CYP2D6* genotype to the base model led to a marked model fit improvement (ΔOFV − 287 points, 6 *d.f., p* < 0.001). The CYP2D6*2 allele parameter was further distinguished into significantly different values based on the rs5758550 variant, further improving the model fit (ΔOFV − 7.8, *p* = 0.005). Moreover, the *NFIB*-C variant allele was significantly associated with increased CYP2D6-mediated clearance (ΔOFV − 6.8, *p* = 0.009), while CYP3A4*22 was not significant as a covariate in the model (*p* = 0.31).

For the impact of aging on the pharmacokinetics of risperidone and 9-hydroxyrisperidone, the exploratory analysis indicated stable clearances of both risperidone and 9-hydroxyrisperidone up to approximately 40 years followed by a linear decline at higher ages. The impacts of age on the clearances were therefore incorporated as piecewise linear functions with estimation of the age breaking points and the extents of linear decline above the age breaking point (Table 2). These functions were supported for 9-hydroxyrisperidone clearance (ΔOFV − 62, *p* < 0.001) and for risperidone clearance (ΔOFV − 25, *p* < 0.001) and were superior to alternative candidate functions. No significant relationship was identified related to patient sex (*p* ≥ 0.23), and no covariates influenced the volume of distributions. After completing the forward inclusion of covariates, each covariate (including each *CYP2D6* variant allele) was removed from the final model sequentially and all were found to contribute significantly to the final model (*p* < 0.01).

**Table 2:** Final model parameter estimates

**Model parameter**	**Parameter estimate**	**Bootstrap median**	**Bootstrap 95% CI**
Risperidone (parent)			
Fixed effects			
Absorption rate constant (k_a_) (h^−1^)	2.01 (fix)^a^		
Volume of distribution (V/F) (L)	333	317	177, 586
Clearance (CL/F) (L/h)			
Base	4.2	4.2	3.6, 4.9
CYP2D6*1	23.2	22.5	15.4, 31.6
*CYP2D6* allele (fraction of CYP2D6*1)			
*2-rs5758550*G*	0.66	0.66	0.50, 0.85
*35	0.57	0.57	0.28, 0.90
*2-rs5758550*A*	0.30	0.31	0.17, 0.49
*9	0.39	0.40	0.20, 0.68
*10	0.32	0.33	0.15, 0.56
*41	0.15	0.16	0.08, 0.26
*3-*6	0 (fix)	-	-
*NFIB* allele (fraction of *NFIB-T*)			
*NFIB-C*	1.41	1.41	1.15, 1.73
Age breaking point (years)	34	34	28, 38
Reduction per year (≥ breaking point)	− 0.9%	− 0.9%	− 1.2%, − 0.5%
Random effects			
BSV CL/F (% CV)	86%	84%	69%, 103%
Residual error, proportional (%)	58%	58%	53%, 62%
Residual error, additive (ng/mL)	0.06	0.06	0.04, 0.15
9-hydroxyrisperidone (metabolite)			
Fixed effects			
Volume of distribution (*V*_met_/F × *f*_met_)	96	97	77, 127
Clearance (CL_met_/F × *f*_met_)	8.0	8.0	7.5, 8.6
Age breaking point (years)	39	39	30, 48
Reduction per year (≥ breaking point)	− 1.3%	− 1.3%	− 1.6%, − 1.0%
Random effects			
BSV CL_met_/*f*_met_ (% CV)	49%	49%	43%, 55%
Residual error, proportional	38%	38%	35%, 41%
Correlation with BSV CL/F	0.43	0.41	0.25, 0.52

Bootstrap-derived values are based on 5000 non-parametric bootstrap replicates. Final clearance functions: CL_risperidone_ = (CL_base_ + [CL_CYP2D6, allele1_ + CL_CYP2D6, allele2_] × CL_NFIB_ [if *CC* or *CT*]) × (1–CL_age_ × [Age–Age_BP_]) [if Age ≥ Age_BP_]; CL_9-hydroxyrisperidone_ = CL × (1–CL_age_ × [Age– Age_BP_]) [if Age ≥ Age_BP_] where each parameter refers to corresponding parameters in the table; Age is patient age, Age_BP_ is the age breaking point, and CL_age_ is the estimated reduction per year

*CL* clearance, *V* volume of distribution, *F* bioavailability, *f*_*met*_ fraction metabolized, *BSV* between-subject variability, *CV* coefficient of variation

^a^Fixed to value from literature [31]

#### Final model evaluation and interpretation

The final model parameters are shown in Table 2. The estimated values were in close agreement with the bootstrap-derived median value for each parameter, and the 95% confidence intervals for each covariate excluded the null values (i.e., values representing no effect).

**Fig. 1:**
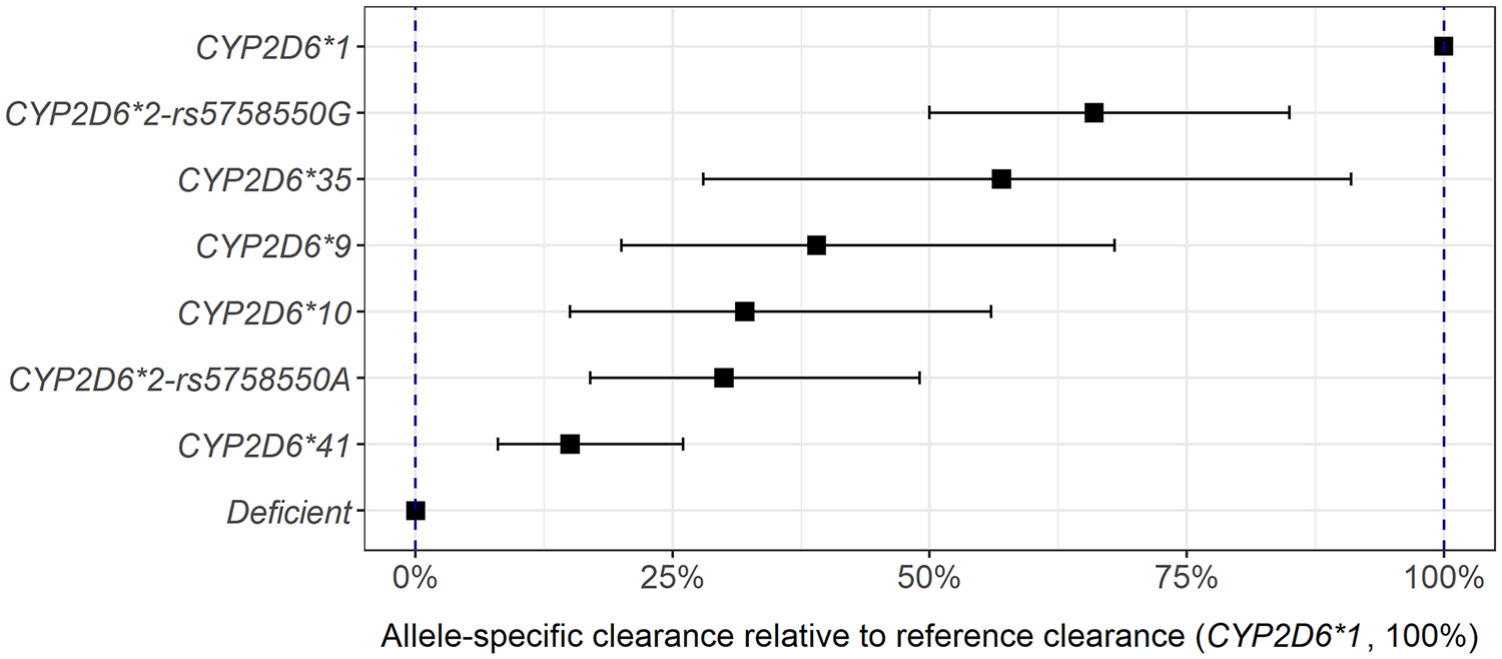
Population model-estimated risperidone clearance for each allele or haplotype, relative to CYP2D6*1. Points represent model estimates, and horizontal lines represent 95% confidence intervals. Each estimate is based on the following number of alleles: CYP2D6*1: 401, CYP2D6*2-rs5758550G: 166, CYP2D6*35: 54, CYP2D6*9: 20, CYP2D6*10: 23, CYP2D6*2-rs5758550A: 27, CYP2D6*41: 66, and deficient (CYP2D6*3, *4, *5, *6): 269

The clearances attributable to the alleles or haplotypes of primary interest were estimated at 30% for CYP2D6*2-rs5758550A, 66% for CYP2D6*2-rs5758550G, and 57% for CYP2D6*35, relative to the clearance for *1 (Fig. 1). For the remaining decreased function alleles *9, *10, and *41, the allele-specific clearances were estimated to be 39%, 32%, and 15%, respectively, relative to the clearance for *1. Homo- or heterozygous carriers of the *NFIB-C* variant were estimated to have 41% higher CYP2D6-mediated clearance compared with subjects with the same *CYP2D6* genotype and *NFIB*-*T*. Finally, aging was associated with a reduction in clearance of 0.9% and 1.3% for each year above the ages of 34 and 39 years for risperidone and 9-hydroxyrisperidone, respectively.

**Fig. 2:**
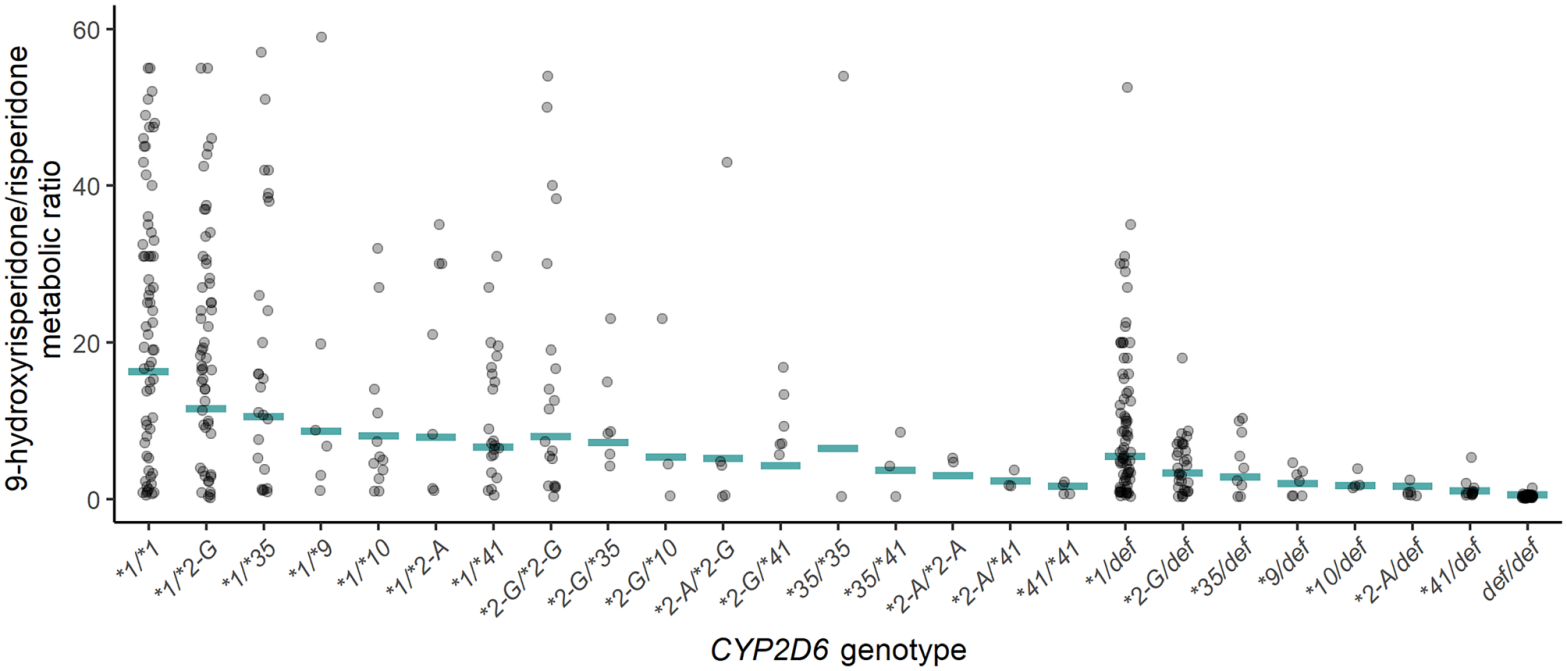
Model-predicted metabolic ratio (9-hydroxyrisperidone/risperidone concentration) together with observed metabolic ratio within each *CYP2D6* diplotype. Horizontal lines represent the model-predicted metabolic ratio in a typical subject (with characteristic values similar to the median in the dataset: age 37 years, *NFIB *genotype* TT*, sampling 13 h after dose). Each dot represents one subject. The median individual values are depicted in the case of multiple observations per subject. Only diplotypes with ≥ 2 subjects are displayed (*n* = 504). Def = deficient allele (*3, *4, *5, or *6). *2-G = CYP2D6*2-rs5758550G. *2-A = CYP2D6*2-rs5758550A

The prediction-corrected visual predictive check (Supplementary Fig. S2) and diagnostic plots (Supplementary Fig. S3) indicated that the model described the mean tendencies and variabilities in the time courses of drug concentrations well for both risperidone and 9-hydroxyrisperidone. The model-predicted metabolic ratio within each *CYP2D6* diplotype was also in overall agreement with the observed metabolic ratios (Fig. 2), supporting that the model captures the trends in the underlying raw data.

### Haplotypes associated with rs5758550 G>A

LD analysis identified 24 SNPs in high LD to rs5758550 (Supplementary Fig. S4 and S5 [39]). These SNPs, together with the SNPs identifying CYP2D6*2, CYP2D6*35, and CYP2D6*41, revealed a total of 34 haplotypes, and among these, 22 haplotypes were rare. The frequencies of CYP2D6*2-rs5758550G and CYP2D6*2-rs5758550A in the European reference cohort were 18.4% and 1.2%, respectively [39]. Within the CYP2D6*2-rs5758550G haplotype, five different sub-haplotypes were found, and the most abundant among these had a frequency of 15.8%. This common haplotype contained all the 24 SNPs that were in high LD with rs5758550 (G>A). Among the CYP2D6*2-rs5758550A haplotypes, the most common sub-haplotype carried only 3 of the SNPs that were in high LD with rs5758550, indicating that 21 of the SNPs are specific for the CYP2D6*2-rs5758550G haplotype. All these SNPs are located downstream of the *CYP2D6* gene (chr22 42022906–chr22 41990945 (136 kb distance)). There is a high degree of interethnic differences for the haplotypes. In the East Asian reference cohort of the 1000 Genomes Project (EAS, *n* = 504), the allele frequency of the CYP2D6*2-rs5758550G and CYP2D6*2-rs5758550A haplotypes was 7.1% and 3%, respectively [39].

## Discussion

Understanding the relationships between *CYP2D6* variants or haplotypes and CYP2D6 activity is important to predict the individual dose requirement for a vast number of drugs. Our study indicates a significant inhibitory effect of the CYP2D6*2 and CYP2D6*35 alleles on risperidone CYP2D6-mediated clearance. Risperidone clearance attributable to the rare CYP2D6*2-rs5758550A haplotype was only 30% relative to the clearance attributable to CYP2D6*1, which is different as compared to the common CYP2D6*2-rs5758550G haplotype where 66% of the clearance was estimated. CYP2D6*2 is recommended for inclusion in *CYP2D6* genotyping panels [13] but is currently classified with similar effects on enzyme activity as the wild type (*1) [3]. If our results also apply to other CYP2D6 substrates, interpreting CYP2D6*2 as fully functional will overpredict drug clearance and dose requirements, especially for CYP2D6*2-rs5758550A carriers.

The association between rs5758550 and increased *CYP2D6* expression was first described by Wang et al., and they hypothesized that this effect and the reduced CYP2D6 activity function encoded by CYP2D6*2 might have opposing effects on phenotype [14, 15]. Subsequent in vivo studies have been conflicting as to whether rs5758550 assessment can improve the prediction of CYP2D6 activity [17–19, 40, 41]. In all these studies, linkage disequilibrium with other SNPs in the locus was not considered, so we investigated the haplotypes carrying the rs5758550 mutation in more detail. We found 24 SNPs that showed high LD with rs5758550. The CYP2D6*2-rs5758550G haplotype included all 24 SNPs in high LD with rs5758550 (G>A), while the CYP2D6*2-rs5758550A haplotype carried only 3 of the SNPs in high LD. CYP2D6*2-rs5758550G was common (frequency 18.4%), while CYP2D6*2-rs5758550A was rare in Europeans (1.2%). However, in East Asians, CYP2D6*2-rs5758550A frequency was 3% [39]. Previously, GWAS analyses of patients taking tamoxifen revealed a high degree of influence of the SNPs on Chr 22 in a region of 400 kb flanking the *CYP2D6 *gene on endoxifen levels [41]. The data suggest that the *CYP2D6* locus on 22q13 within this 400 kb region is the most important genetic determinant for the regulation of *CYP2D6* expression. Since the specific SNPs in the non-CYP2D6 coding regions in this locus have not yet been identified, it is not possible to clarify which of the SNPs in high LD to CYP2D6*2-rs5758550 might be important for the regulation of *CYP2D6* gene expression. This complexity may explain the contradictory results of previous studies, which are due to incomplete characterization of the alleles and also impair the current potential clinical use of this finding.

In our analysis, we also estimated risperidone CYP2D6-mediated clearance attributable to the CYP2D6*35 allele to be 57% compared with CYP2D6*1, based on a total sample of 54 CYP2D6*35 alleles. This estimate is similar to the previously reported value of 46% enzyme activity for the CYP2D6*35 allele in a population pharmacokinetic study on carvedilol [8]. For tamoxifen, reduced activity for the *35 allele has been reported in vitro [12], but not in vivo [6], suggesting potential substrate dependency.

The impacts of the well-known reduced function alleles CYP2D6*9, *10, and *41 on risperidone CYP2D6-mediated clearance were also estimated in our analysis. The largest activity reduction was estimated for CYP2D6*41 (15% residual activity), while 39% and 32% residual activities were estimated for CYP2D6*9 and *10, respectively. These impacts are as expected and in line with previous reports [7, 42], which also add confidence to the estimated effects associated with less studied variant alleles CYP2D6*35 and CYP2D6*2 as well as the rs5758550 SNP. Furthermore, we estimated that the impact of the mutated *NFIB-C* variant causes 41% increased CYP2D6-mediated clearance compared with the same *CYP2D6* allele and *NFIB-T*. This is in line with our previous findings in a partly overlapping patient sample [29].

The clearance of risperidone in each subject was modeled as the sum of the partial clearances attributable to each individual *CYP2D6* allele, added to the CYP2D6-independent clearance term. In a typical normal metabolizer (e.g., CYP2D6 *1/*1), risperidone clearance is predicted to be 50.6 L/h. In a typical intermediate metabolizer (e.g., CYP2D6 *1/deficient allele), risperidone CL/F is predicted to be 27.4 L/h, whereas in a poor metabolizer (two *CYP2D6* deficient alleles), risperidone CL/F is predicted to be 4.2 L/h. These values are similar to the reported values in previously performed population pharmacokinetic analyses of risperidone in adult subjects with known *CYP2D6* genotypes [43] as well as when *CYP2D6* genetic subpopulations were estimated using mixture modelling [44, 45].

In addition to the genetic covariates, aging was estimated to be associated with a decline in both risperidone and 9-hydroxyrisperidone clearance by 0.9 and 1.3% per year, respectively, after 34 and 39 years of age. The effect of aging on 9-hydroxyrisperidone has been reported previously [31, 43, 44] and is expected since 9-hydroxyrisperidone is renally eliminated and age-related changes in renal function occur between 30 and 40 years of age [46]. However, the precise mechanism underlying the age-related decline in risperidone clearance remains somewhat elusive as CYP2D6 activity remains relatively stable in the elderly. Age-related alterations in hepatic blood flow could play a role as risperidone exhibits intermediate hepatic extraction ratio [47]. The model predicts that the total active moiety of risperidone and 9-hydroxyrisperidone would be more than doubled in subjects 75 years and older compared with a 30-year-old subject and supports the current recommendation of lower risperidone starting doses in the elderly.

Risperidone was selected as a CYP2D6 activity marker in this study to evaluate the impacts of allele variants on CYP2D6-mediated clearance. Notably, the major metabolite 9-hydroxyrisperidone is pharmacologically active, and this limits the clinical relevance of altered risperidone clearance [43]. The Dutch Pharmacogenetic Working Group recommends that CYP2D6 poor metabolizers (i.e., patients carrying two deficient *CYP2D6* alleles) should start on 67% of the standard risperidone dose, while no action is recommended for intermediate metabolizers [48]. Implementation of the reduced function haplotypes studied here would therefore not lead to altered risperidone starting dose recommendations. Thus, our results may be more relevant to optimize treatment with CYP2D6 substrates exhibiting narrower therapeutic indexes, including tricyclic antidepressants, and drugs dependent on CYP2D6 for bioactivation, such as tamoxifen, codeine, and tramadol. Considering the potential substrate-dependent effects of the investigated haplotypes, specific studies for such substances are needed prior to clinical implementation.

A limitation of our study is that the concentration measurements are derived from a TDM setting where measurements typically are only available from one sampling time during the dose interval. Further, information about patient characteristics was limited to age, sex, and genetics, with no information on body size or organ function parameters, which may also impact on risperidone pharmacokinetics. Still, as these missing potential covariates are not likely to be correlated with the genetic variants under investigation, the estimated genetic covariate effects are not expected to be biased. Another limitation is that it was not possible to determine at which allele the rs5758550 variant was located in heterozygous samples (e.g., rs5758550 *A/G* and CYP2D6 *1/*2). In such samples, it was assumed that the diplotype was *1-rs5758550A/*2-rs5758550G, but it cannot be excluded that some of these subjects were truly *1-rs5758550G/*2-rs5758550A carriers. As we only identified three subjects (< 1%) with rs5758550 *A/G* and CYP2D6 *1/*1, we do not believe that merging these subjects with the cohort of subject with rs5758550 *A/A* and CYP2D6 *1/*1 genotype has influenced the results.

To summarize, we used the population pharmacokinetic approach to investigate the effects of *CYP2D6* variant alleles with unclear effects on enzyme activity using risperidone clearance as a CYP2D6 activity marker. Our results demonstrate that CYP2D6*2 and CYP2D6*35 encode reduced risperidone clearance and that there are two different haplotypes of CYP2D6*2 in linkage to rs5758550 associated with different extent of clearance reduction*.* Thus, genotyping of these haplotypes might improve precision of genotype-guided prediction of CYP2D6-mediated clearance.

## Supplementary Information

Below is the link to the electronic supplementary material.

Supplementary Table S11 (DOCX 17 KB)

Supplementary Fig. S1. Raw data exploration of the observed metabolic ratio (9-hydroxyrisperidone/risperidone concentration) within each *CYP2D6* diplotype. The median value depicted in case of multiple observations per subject. The boxes are colored by their previous clinical routine determined *CYP2D6* genotype. def = deficient allele (*3, *4, *5 or *6). Red = reduced function allele (*9, *10, *41). *2-G = CYP2D6*2-rs5758550G. *2-A = CYP2D6*2-rs5758550A. (TIFF 11865 KB)

Supplementary Fig. S2. Prediction-corrected visual predictive check (pcVPC) for the final risperidone and 9-hydroxyrisperidone population pharmacokinetic model (top panels: normal scales, bottom panels: semi-logarithmic scales). Dots represent observed concentration measurements. Red solid line represents median observed concentration. Blue solid lines represent the 5th and 95th percentiles of the observed concentrations, respectively. Red/blue-shaded areas represent 95% confidence interval for the corresponding model-predicted percentiles. (TIFF 16875 KB)

Supplementary Fig. S3. Diagnostic plots for the final risperidone and 9-hydroxyrisperidone population pharmacokinetic model. Top: Population predicted vs. observed concentration; Bottom: Time after dose vs. conditional weighted residual (CWRES). (TIFF 12656 KB)

Supplementary Fig. S4. Proxies for rs5758550 in European population. The *CYP2D6* gene is located on chromosome 22: 42126499–42130865 reverse strand. Twenty-four SNPs were identified to be in high linkage disequilibrium (LD) to rs5758550 with R^2^ values > 0.85. Figure from LD-link [39]. (JPEG 1060 KB)

Supplementary Fig. S5. Haplotypes CYP2D6*2-rs5758550G and CYP2D6*2-rs5758550A. The most common subvariants of each haplotype are presented. The two SNPs representing CYP2D6*2, rs16947 (exon 6) and rs1135840 (exon 9), are indicated to the right. The rs5758550 G>A SNP is also indicated. The rest of the nucleotides in the CYP2D6*2-rs5758550G haplotype (upper part) represent the 24 SNPs in high LD with rs5758550 G>A. The three SNPs that are shared with CYP2D6*2-rs5758550A are indicated with vertical lines. The other 21 SNPs are unique for the CYP2D6*2-rs5758550G haplotype vs CYP2D6*2-rs5758550A. The 24 SNPs in high linkage disequilibrium with rs5758550 G>A are located within 136 kb just downstream of the *CYP2D6* gene (chromosome 22: 42126499–42130865 reverse strand). The figure was made using Biorender.com. (TIF 7012 KB)

## Data Availability

No datasets were generated or analysed during the current study.
