# Supplementary material for: Impact of CYP2D6*2, CYP2D6*35, rs5758550, and related haplotypes on risperidone clearance in vivo
Source: Eur J Clin Pharmacol. 2024 Jul 4;80(10):1531–41. doi: 10.1007/s00228-024-03721-6 (PMC11393095; doi:10.1007/s00228-024-03721-6)
Supplement: Supplementary file 1 — Supplementary Table S11 (DOCX 17 KB) [file 228_2024_3721_MOESM1_ESM.docx]

**Supplementary Table S1.**

Overview of subjects, samples and raw 9-hydroxyrisperidone/risperidone metabolic ratio for each *CYP2D6* diplotype

| *CYP2D6 diplotype* | Subjects (observations), n | *Metabolic ratio*  *Median* | *Pairwise comparison reference group* | *p* |
| --- | --- | --- | --- | --- |
| **1/*1xN* | 1 | - |  |  |
| **2/*2xN* | 1 | - |  |  |
|  |  |  |  |  |
| **1/*1* | 82 (218) | 25.5 |  |  |
|  |  |  |  |  |
| **1/*2-G* | 62 (156) | 18.7 | **1/*1* | 0.06 |
| **2-G/*2-G* | 20 (64) | 12.1 | **1/*2-G* | 0.27 |
|  |  |  |  |  |
| **1/*2-A* | 7 (12) | 21.0 | **1/*1* | 0.22 |
|  |  |  | **1/*2-G* | 0.68 |
| **2-A/*2-A* | 2 (2) | 5.0 | *-* | - |
| **2-A/*2-G* | 6 (9) | 4.6 | **2-G/*2-G* | 0.15 |
|  |  |  |  |  |
| **1/*35* | 30 (115) | 15.7 | **1/*1* | 0.18 |
| **35/*35* | 2 (2) | 27.1 | *-* | - |
| **2-G/*35* | 6 (41) | 8.5 | **1/*35* | 0.22 |
|  |  |  | **1/*2-G* | 0.12 |
|  |  |  |  |  |
| **1/*9* | 8 (16) | 7.8 | **1/*1* | 0.03 |
| **2-G/*9* | 1 (2) | 11.0 | *-* |  |
| **9/*9* | 1 (2) | 2.9 | *-* |  |
| **9/*35* | 1 (6) | 7.8 | *-* |  |
| **9/*41* | 1 (1) | 2.8 | *-* |  |
|  |  |  |  |  |
| **1/*10* | 13 (44) | 5.4 | **1/*1* | 0.002 |
| **2-G/*10* | 3 (7) | 4.5 | **1/*10* | 0.61 |
| **10/*10* | 1 (7) | 9.0 | *-* |  |
| **10/*35* | 1 (1) | 7.3 | *-* |  |
|  |  |  |  |  |
| **1/*41* | 26 (66) | 8.0 | **1/*1* | <0.001 |
| **2-G/*41* | 8 (48) | 8.2 | **1/*41* | 0.87 |
| **2-A/*41* | 3 (6) | 1.8 | **1/*41* | 0.06 |
|  |  |  | **2-G/*41* | 0.02 |
| **35/*41* | 3 (8) | 4.2 | **1/*41* | 0.17 |
| **41/*41* | 5 (10) | 1.3 | **1/*41* | 0.001 |
|  |  |  |  |  |
| **1/def* | 88 (274) | 7.0 | **1/*1* | <0.001 |
| **2-G/def* | 37 (107) | 3.8 | **1/def* | 0.002 |
| **2-A/def* | 7 (13) | 0.8 | **1/def* | <0.001 |
|  |  |  | **2-G/def* | 0.003 |
| **35/def* | 9 (25) | 4.0 | **1/def* | 0.12 |
| **9/def* | 7 (27) | 2.3 | **1/def* | 0.004 |
| **10/def* | 4 (13) | 1.7 | **1/def* | 0.08 |
| **41/def* | 15 (49) | 0.8 | **1/def* | <0.001 |
| *def/def* | 51 (214) | 0.3 | **1/def* | <0.001 |

**2-A* refers to haplotype *CYP2D6*2*-rs5758550*A* and **2-G* refers to haplotype *CYP2D6*2*-rs5758550*G*.

def=deficient allele (**3, *4, *5* or **6*).

The pairwise comparisons were performed using Mann-Whitney U test.
