# Supplementary figures and images for: Impact of CYP2D6*2, CYP2D6*35, rs5758550, and related haplotypes on risperidone clearance in vivo
Source: Eur J Clin Pharmacol. 2024 Jul 4;80(10):1531–41. doi: 10.1007/s00228-024-03721-6 (PMC11393095; doi:10.1007/s00228-024-03721-6)

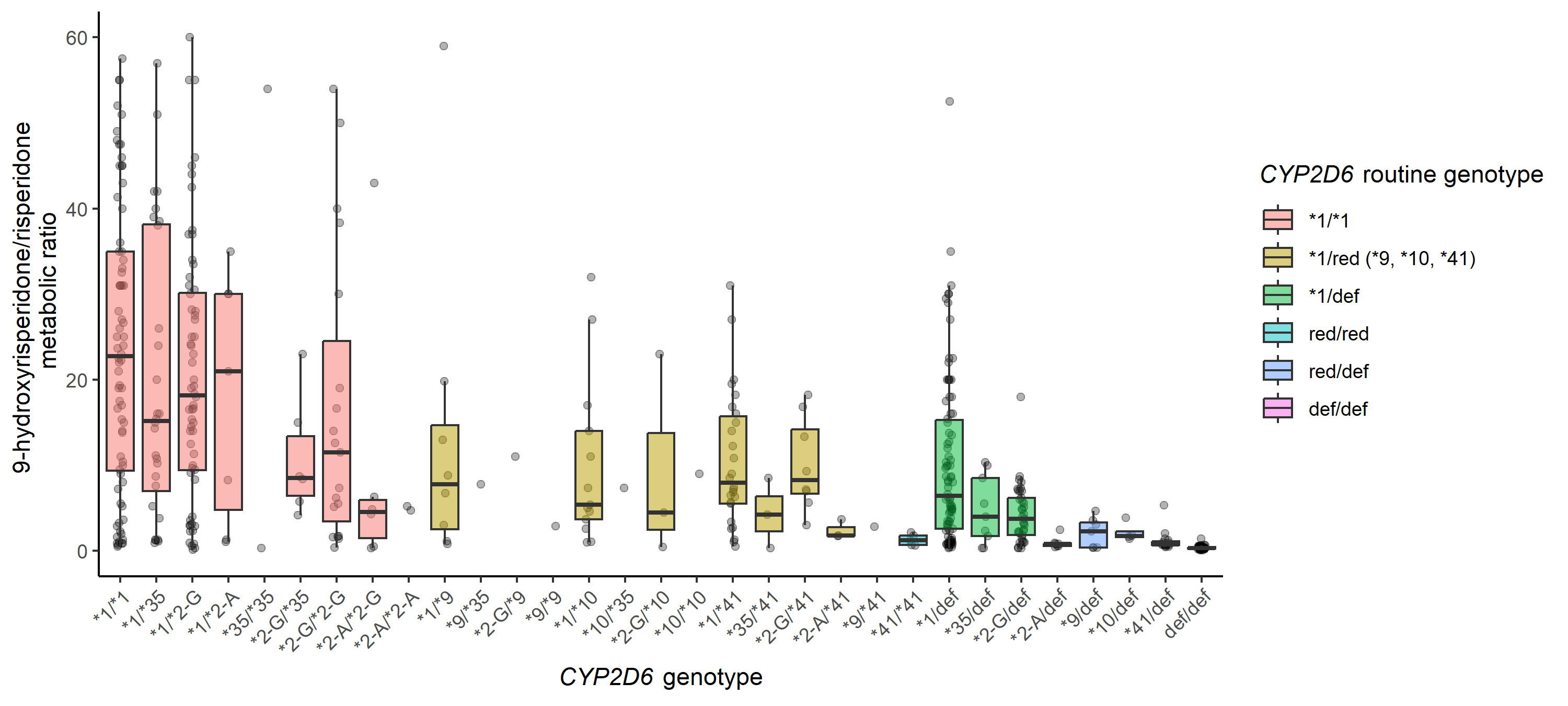

Supplement: Supplementary file 2 — Supplementary Fig. S1. Raw data exploration of the observed metabolic ratio (9-hydroxyrisperidone/risperidone concentration) within each CYP2D6 diplotype. The median value depicted in case of multiple observations per subject. The boxes are colored by their previous clinical routine determined CYP2D6 genotype. def = deficient allele (*3, *4, *5 or *6). Red = reduced function allele (*9, *10, *41). *2-G = CYP2D6*2-rs5758550G. *2-A = CYP2D6*2-rs5758550A. (TIFF 11865 KB) [file 228_2024_3721_MOESM2_ESM.tiff]

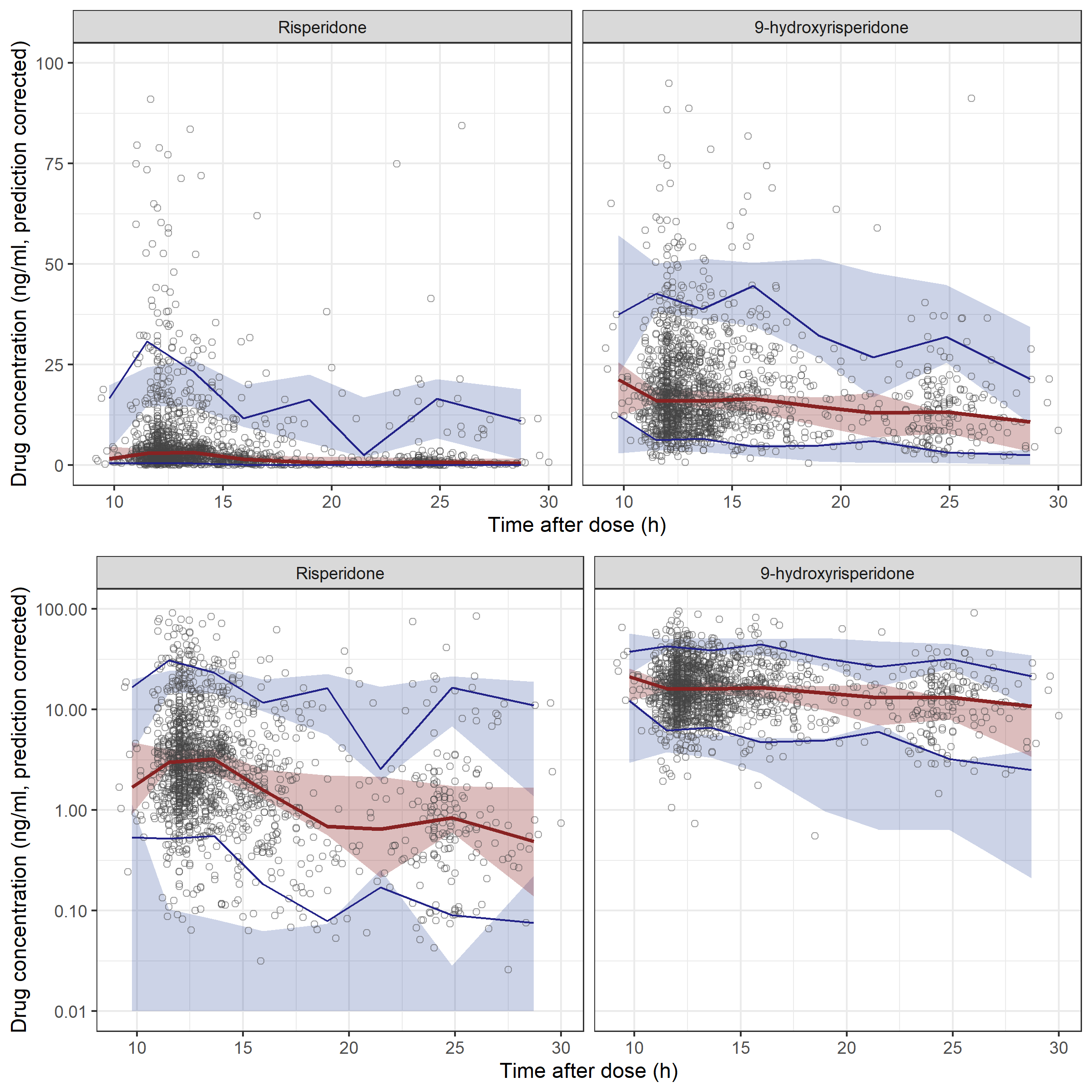

Supplement: Supplementary file 3 — Supplementary Fig. S2. Prediction-corrected visual predictive check (pcVPC) for the final risperidone and 9-hydroxyrisperidone population pharmacokinetic model (top panels: normal scales, bottom panels: semi-logarithmic scales). Dots represent observed concentration measurements. Red solid line represents median observed concentration. Blue solid lines represent the 5th and 95th percentiles of the observed concentrations, respectively. Red/blue-shaded areas represent 95% confidence interval for the corresponding model-predicted percentiles. (TIFF 16875 KB) [file 228_2024_3721_MOESM3_ESM.tiff]

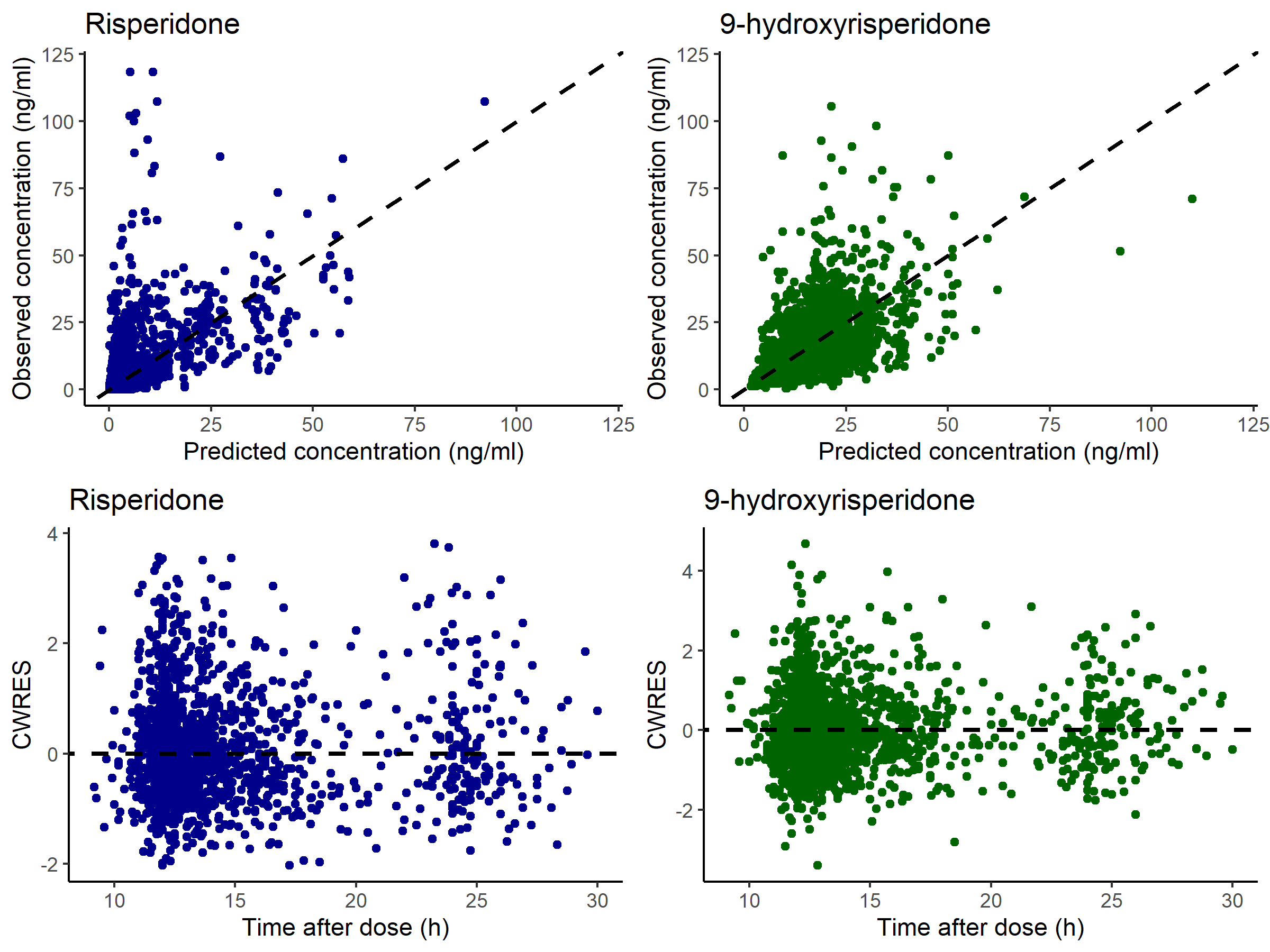

Supplement: Supplementary file 4 — Supplementary Fig. S3. Diagnostic plots for the final risperidone and 9-hydroxyrisperidone population pharmacokinetic model. Top: Population predicted vs. observed concentration; Bottom: Time after dose vs. conditional weighted residual (CWRES). (TIFF 12656 KB) [file 228_2024_3721_MOESM4_ESM.tiff]

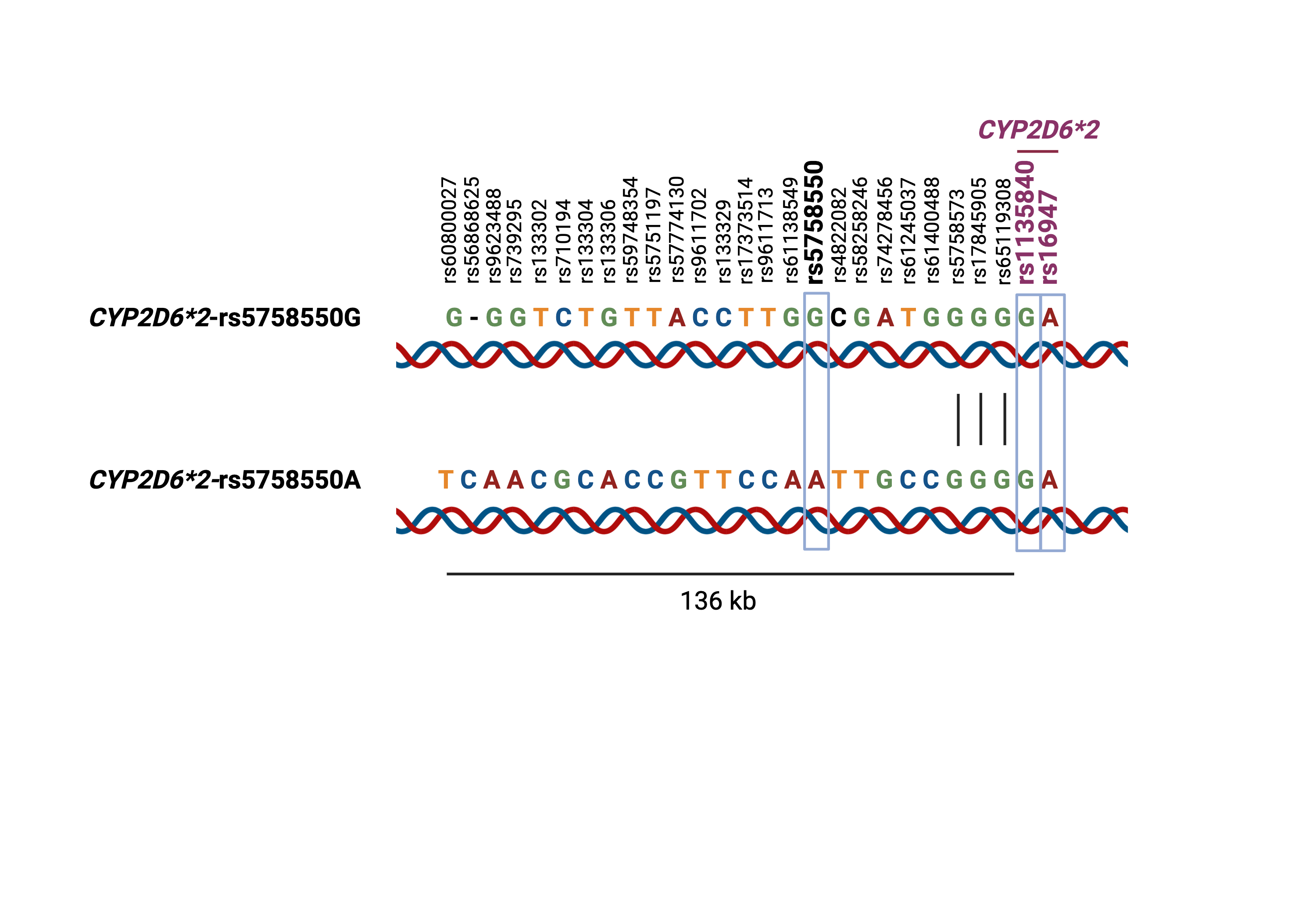

Supplement: Supplementary file 5 — Supplementary Fig. S4. Proxies for rs5758550 in European population. The CYP2D6 gene is located on chromosome 22: 42126499–42130865 reverse strand. Twenty-four SNPs were identified to be in high linkage disequilibrium (LD) to rs5758550 with R2 values > 0.85. Figure from LD-link [39]. (JPEG 1060 KB) [file 228_2024_3721_MOESM5_ESM.jpeg]

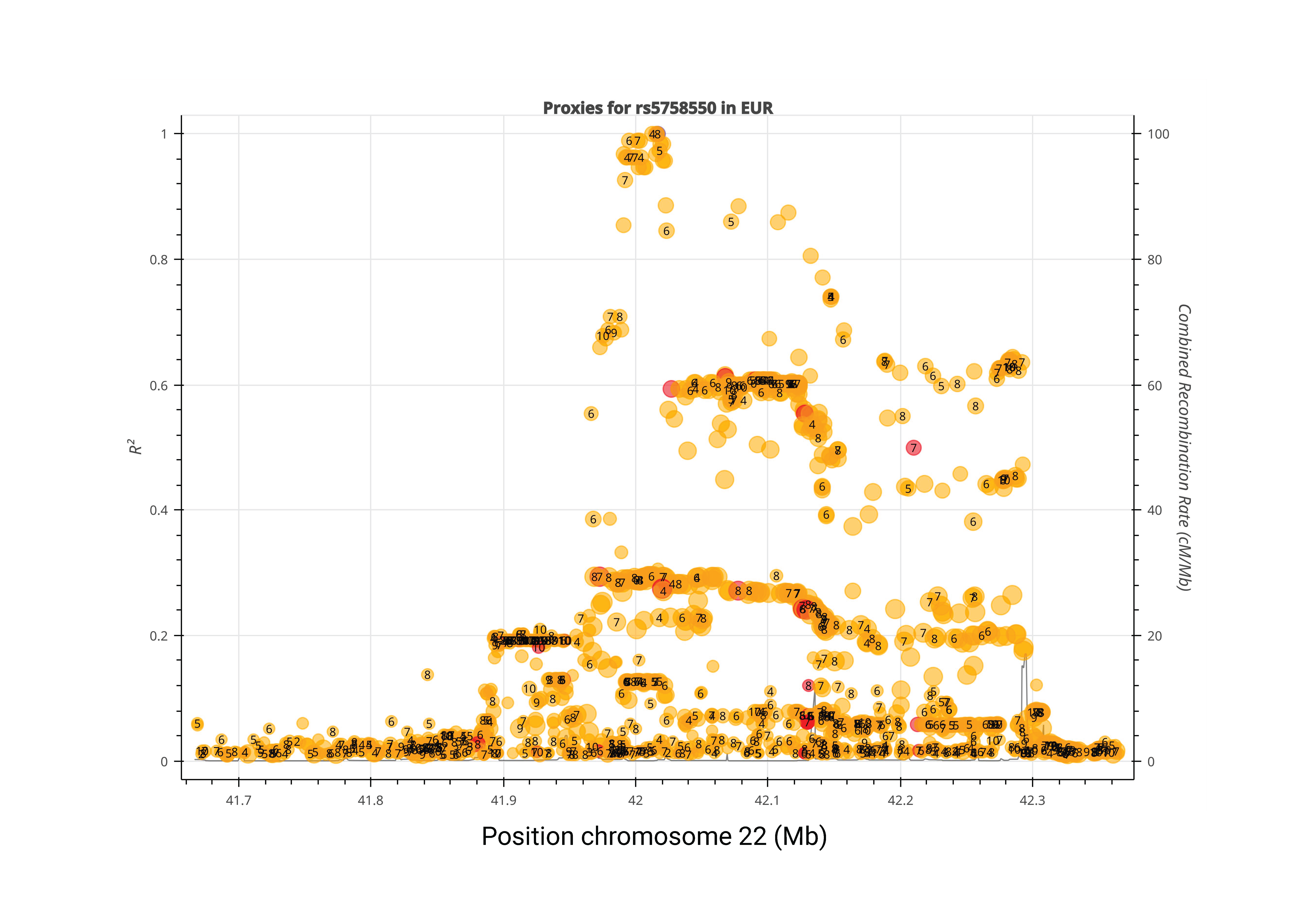

Supplement: Supplementary file 6 — Supplementary Fig. S5. Haplotypes CYP2D6*2-rs5758550G and CYP2D6*2-rs5758550A. The most common subvariants of each haplotype are presented. The two SNPs representing CYP2D6*2, rs16947 (exon 6) and rs1135840 (exon 9), are indicated to the right. The rs5758550 G>A SNP is also indicated. The rest of the nucleotides in the CYP2D6*2-rs5758550G haplotype (upper part) represent the 24 SNPs in high LD with rs5758550 G>A. The three SNPs that are shared with CYP2D6*2-rs5758550A are indicated with vertical lines. The other 21 SNPs are unique for the CYP2D6*2-rs5758550G haplotype vs CYP2D6*2-rs5758550A. The 24 SNPs in high linkage disequilibrium with rs5758550 G>A are located within 136 kb just downstream of the CYP2D6 gene (chromosome 22: 42126499–42130865 reverse strand). The figure was made using Biorender.com. (TIF 7012 KB) [file 228_2024_3721_MOESM6_ESM.tif]
